# Impacts of Duck-Origin Parvovirus Infection on Cherry Valley Ducklings From the Perspective of Gut Microbiota

**DOI:** 10.3389/fmicb.2019.00624

**Published:** 2019-03-28

**Authors:** Qihui Luo, Jing Xu, Chao Huang, Xinyu Lei, Dongjing Cheng, Wentao Liu, Anchun Cheng, Li Tang, Jing Fang, Yangping Ou, Yi Geng, Zhengli Chen

**Affiliations:** ^1^Key Laboratory of Animal Disease and Human Health of Sichuan Province, College of Veterinary Medicine, Sichuan Agricultural University, Chengdu, China; ^2^Laboratory of Experimental Animal Disease Model, College of Veterinary Medicine, Sichuan Agricultural University, Chengdu, China

**Keywords:** D-GPV, BADS, gut microbiota dysbiosis, intestinal inflammation, SCFAs

## Abstract

Duck-origin goose parvovirus (D-GPV) is the causative agent of beak atrophy and dwarfism syndrome (BADS), characterized by growth retardation, skeletal dysplasia, and persistent diarrhea. However, the pathogenic mechanism of D-GPV remains undefined. Here, we first reported the gut microbiome diversity of D-GPV infected Cherry Valley ducks. In the investigation for the influence of D-GPV infection on gut microbiota through a period of infection, we found that D-GPV infection caused gut microbiota dysbiosis by reducing the prevalence of the dominant genera and decreasing microbial diversity. Furthermore, exfoliation of the intestinal epithelium, proliferation of lymphocytes, up-regulated mRNA expression of pro-inflammatory TNF-α, IL-1β, IL-6, IL-17A, and IL-22 and down-regulated mRNA expression of anti-inflammatory IL-10 and IL-4 occurred when D-GPV targeted in cecal epithelium. In addition, the content of short chain fatty acids (SCFAs) in cecal contents was significantly reduced after D-GPV infection. Importantly, the disorder of pro-inflammatory and anti-inflammatory cytokines was associated with the decrease of SCFAs-producing bacteria and the enrichment of opportunistic pathogens. Collectively, the decrease of SCFAs and the enrichment of pathogen-containing gut communities promoted intestinal inflammatory injury. These results may provide a new insight that target the gut microbiota to understand the progression of BADS disease and to research the pathogenic mechanism of D-GPV.

## Introduction

The diverse array of host characteristics can be influenced by the gut microbiota, including nutrition and metabolism, innate and adaptive immunity, mucus composition, proliferation and differentiation of enterocyte, and resistance to pathogen colonization (Hooper et al., 2012; Tremaroli and Bäckhed, 2012; Kim et al., 2016). There are several billions of bacteria, including pathogenic and non-pathogenic species, the commensal and the opportunistic bacteria, present in poultry intestines where is one of the primary sites of exposure to pathogens (Adegunloye, 2006). The commensal bacteria could cause pathology after translocation through the mucosa or under the condition of immunodeficiency, even though most of the bacteria are symbiotic (Marchetti et al., 2008; Glavan et al., 2016). Significant shifts in the microbiota are one of the characteristics of acute mucosal infections, a phenomenon known as dysbiosis (Spor et al., 2011). Furthermore, the infections of gastrointestinal tract can also promote expansion of commensal bacteria with pro-inflammatory potential which can directly exacerbate the pathological process (Egan et al., 2011). Thus, infections can threaten the homeostatic relationship between the host and its gut microbiota in this highly reactive environment. Considering the potential relationships between the gut microbiota and viral infection (Wilks and Golovkina, 2012; Lynch, 2014) and the vital roles of the gut microbiota in regulating the immunity and inflammatory disease (Nobuhiko et al., 2013; Sun et al., 2015). It is vitally important to understand how the composition of the gut microbiota alters as a consequence of Duck-origin Parvovirus (D-GPV) infection and the meanings of these alterations on the pathogenic mechanism of pathogen.

Duck-origin Parvovirus, a novel Goose Parvovirus, is the pathogen of the beak atrophy and dwarfism syndrome (BADS), and has been emerging as a severe threat to the health of duck flocks since 2015 in China (Chen et al., 2015). D-GPV was highly pathogenic to mule ducks and Cherry Valley ducks, and all experimentally infected ducks exhibited atrophic beak, projecting tongue, remarkable growth retardation, skeletal dysplasia and diarrhea similar to naturally infected ducks (Chen H. et al., 2016; Ning et al., 2018). Due to the significant growth retardation, BADS has caused great economic losses to China. Here, we reported an isolate of D-GPV (QH-L01) isolated from Cherry Valley ducks with BADS, a distinct GPV variant strain, which was most closely related to SYG26-35 GPV strains (Chen et al., 2017). Notably, the gastrointestinal abnormalities of diarrhea, weight loss and malnutrition have been observed in BADS ducks. However, the reason of gastrointestinal abnormalities symptoms caused by D-GPV infection is still unclear. Furthermore, there are currently no researches on how D-GPV affects gut microbiota or intestinal health.

To date, the influence of D-GPV infection on gut microbiota of Cherry Valley ducks has not been previously reported. In this study, the cecal microbiota of Cherry Valley ducks were analyzed in order to determine whether potential associations exist between the gut microbiota and gastrointestinal abnormalities after D-GPV infection in a typical industry growth period. Our study would provide the initial basis for understanding the interaction between D-GPV and gut microbiota of Cherry Valley duck.

## Materials and Methods

### Duck-Origin Parvovirus and Experimental Animals

The D-GPV strain, QH-L01, was originally isolated from the liver of the Cherry Valley duckling flock with BADS in Sichuan province, China (Chen et al., 2017). Forty 2-day-old Cherry Valley ducklings free of specific maternal antibodies were obtained from the breeding facility of the Institute of Poultry Sciences in Sichuan Agricultural University, China. Referring to our previous study (Chen et al., 2017), the titer of QH-L01 was calculated at 10^6.54^ EID_50_/0.2 mL for intramuscular challenge.

### Animal Experiments

Forty Cherry Valley ducklings, 2-day-old and free from specific maternal antibodies, were randomly allocated into infection group and control group, housed in isolated animal houses and supplied commercial forage of ducklings (TONGWEI Co., Ltd., China) and water *ad libitum*. The commercial forage for ducklings mainly included 20% protamine, 3.9% crude fiber, 1.1% calcium, and 0.5% phosphorus as detailed in Supplementary Table S1. Twenty ducklings of infection group were inoculated with D-GPV QH-L01 at 10^6.54^ EID_50_/0.2 mL through intramuscular injection at 2 days of age. Twenty ducklings of control group were inoculated with an equivalent volume of sterile phosphate buffered saline (PBS). Daily clinical symptoms and body weight were monitored, and ten ducklings from infection and control groups were euthanized and autopsied at 6 and 15 days post infection (dpi) until the experiments were completed, respectively. Animal experimentation protocols were approved by the Institutional Animal Care and Use Committee of Sichuan Agricultural University, following the guidelines on animal experiments under the permit No.DY-S20164037.

### Sample Collection

All infected ducklings were identified as positive infection by PCR analysis of cloacal swabs as described in our previous study (Luo et al., 2019). The unilateral caeca (cecal tissue and contents) of each animal were removed aseptically, and stored at -80°C until they were processed for total cecal microbial DNA analyses. The one-half of another unilateral caeca tissue and contents were collected and stored at -80°C for RNA isolation and the analysis of short-chain fatty acids (SCFAs). The remaining half of unilateral caeca samples from each ducks was fixed in 4% paraformaldehyde and embedded in paraffin wax, then cut into 5 μm thick sections. Sections were stained with hematoxylin and eosin (H&E) and detected virus antigen with immunohistochemical (IHC) staining.

### Bacterial DNA Isolation

Bacterial DNA was extracted for broad-range amplification and sequence analysis of bacterial 16S rRNA genes as detailed in Supplementary Materials. In brief, bacterial DNA was extracted from cecum and its contents using the Fast DNA SPIN extraction kits (MP Biomedicals, Santa Ana, CA, United States), following the manufacturer’s instructions. The quantity and quality of extracted DNAs were measured using a NanoDrop ND-1000 spectrophotometer (Thermo Fisher Scientific, Waltham, MA, United States) and agarose gel electrophoresis, respectively.

### Bacterial 16S rRNA Gene Sequencing

Bacterial communities of cecal contents and mucosa-adherent bacteria were determined by Illumina MiSeq sequencing of 16S rRNA genes V3–V4 region, and the caeca samples were collected from D-GPV-infected ducks and healthy control ducks at 6 dpi and 15 dpi, respectively. The bacterial V3–V4 region of 16S rRNA genes was amplified using the forward primer 338F (5′-ACTCCTACGGGAGGCAGCA-3′) and the reverse primer 806R (5′-GGACTACHVGGGTWTCTAAT-3′). After purifying the PCR amplicons followed by the quantification of DNA concentration, amplicons were pooled in equal amounts, and pair-end 2 × 300bp sequencing was performed using the Illlumina MiSeq platform with MiSeq Reagent Kit v3 at Shanghai Personal Biotechnology Co., Ltd (Shanghai, China). The sequencing data were deposited into the Sequence Read Archive (SRA) of NCBI (Accession No. SRP182571). The Quantitative Insights Into Microbial Ecology (QIIME, v1.8.0) pipeline was employed to process the sequencing data, as previously described (Caporaso et al., 2010). After quality filters, the remaining high-quality sequences were clustered into operational taxonomic units (OTUs) at 97% sequence identity by UCLUST and as detailed in Supplementary Materials.

### Determination of SCFAs in Cecum

The SCFAs of cecal contents were extracted by the method as described by Baere et al. (2013) with slight modification. Weight 1 g of cecal contents samples were used to dilute at ratio 1:4 to 1:8 (w/v) in sterile distilled water. After vortex mixing for 1 min followed by centrifuge at 5,000 ×*g* for 10 min, the SCFAs-containing supernatant was filtered through cellulose acetate membrane with a pore size of 0.22 μm. SCFAs analyses were carried out by using HPLC as described by Hudafaujan et al. (2010) method.

### Immunohistochemical and Histopathological Analyses

All procedures of IHC staining were performed following the previously described protocols (Chen et al., 2009). Rabbit monoclonal antibody against GPV VP3 protein (Beijing Bioss Biotechnology Co., Ltd., China) was diluted in 1:200, as the primary antibody. After incubating with the primary antibody overnight at 4°C followed by washing three times with PBS, the sections were incubated with mouse anti-rabbit secondary antibody (Biotin-Streptavidin HRP Detection Systems, Boster Biological Technology Co., Ltd., China) for 30 min at 37°C. The positive staining cells appeared dark-brown, denoting the existence of D-GPV antigen, while the negative staining cells were blue under light microscope (Nikon 80i). Caeca samples of the proximal ileum were concurrently stained with H&E prior to catching under a light microscope to assess the histopathology. Ten complete structures of the villus height and crypt depth of every sample were measured using Image-Pro Plus 6.0 (Media Cybernetics, Silver Spring, MD, United States), and the villus height and crypt depth (V/C) ratios were calculated.

### RNA Preparation and Quantitative RT-PCR

Total RNA was isolated from selected cecum using RNAiso Plus reagent [Takara Biomedical Technology (Beijing) Co., Ltd]. Complementary DNA (cDNA) was synthesized using PrimeScript^TM^ RT reagent Kit with gDNA Eraser (Perfect Real Time) [Takara Biomedical Technology (Beijing) Co., Ltd] in accordance with the manufacturer’s instructions. Real-time PCR reactions was performed with final concentrations of 2 × TB Green Premix DimerEraser [Takara Biomedical Technology (Beijing) Co., Ltd], and 0.3 μM forward and reverse primers in 25 μL, using the following conditions: 95°C for 30 s; 40 cycles of 95°C (5 s) and 60°C (30 s) in the Bio-Rad CFX96 Real-Time Detection System (Bio-Rad, United States). The PCR products were detected and monitored by direct measurement of the fluorescence intensity. All measurements were performed in triplicate.

For analysis, target gene expression of each sample was normalized to the house-keeping gene, glyceraldehyde-3-phosphate dehydrogenase (GAPDH), following a previously described protocol (Nair et al., 2011). The 2^-ΔΔCT^ method was used to analyze the results of the qPCR. Sequences of primers used for qRT-PCR specific for cytokines of ducks, were shown in Table 1, including pro-inflammatory TNF-α, IL-1β, IL-6, IL-17A, and IL-22, as well as anti-inflammatory IL-10 and IL-4. Results were expressed as mean ± SEM.

**Table 1:** List of primers used in this study and their sequences.

Gene	Primers 5′ -3′	Accession	Product (bp)
GAPDH	F:TGCTTGCTGCTCCTCCTCAT	GU564233.1	110
	R:TGGCTACCACTTGGACTTTGC		
TNF-α	F:CGTTGACTTGGCTGTCGTGTG	AY765397.1	101
	R:GTGTTCCACATCTTTCAGAGCATC		
IL-1β	F:GCTACACCCGCTCACAGTCCTT	DQ393268.1	123
	R:GCCTCACTTTCTGGCTGGATG		
IL-6	F:TCTGGCAACGACGATAAGGC	XM_027450925.1	156
	R:AATGAAGTAAAGTCTCGGAGGATGA		
IL-17A	F:ACCCTTCGTGCTTCTCTGTC	EU366165.1	155
	R: GCATCTTTTTGGGTCAGGCA		
IL-22	F: TTCCTGGCATCCCTGACCTC	XM_013196285.1	123
	R: ATTCTTTCCATTCTCTCCCAACTGT		
IL-10	F: GACGGGAAACCCAAGTGACA	NM_001310368.1	124
	R: CCTTGATGGAGCCCCTCATT		
IL-4	F: TGCAGGCAATGAGACAGG	MF346730.1	113
	R: GCAGCAAGTTGAGGTAGATG		

### Statistical and Bioinformatics Analysis

The SPSS software package 20.0 (SPSS v. 16, SPSS Inc., Chicago, IL, United States) was used for the statistical calculations in this study. After confirming the normal distribution of variables by the Shapiro–Wilk test, two-tailed Student’s *t*-test and the *post hoc* ANOVA was performed to analyze the variables of normal distribution, and means were considered significantly different at a *P* < 0.05, and summary of *P*-values for multiple comparisons are presented as *P* < 0.01 and *P* < 0.05. When data was not normally distributed it was log-transformed.

The QIIME (v1.8.0) and R packages (v3.2.0) were employed to process the sequence data. OTU-level alpha diversity indices, such as Chao1 richness estimator, ACE metric, Shannon diversity index, and Simpson index, were calculated using the OTU table in QIIME (Hughes et al., 2001). The beta diversity analysis, which utilized UniFrac distance metrics to investigate the structural variation of microbial communities across samples, and visualized via principal coordinate analysis (PCoA) and unweighted pair-group method with arithmetic means (UPGMA) hierarchical clustering, was conducted with R software. LEfSe (Linear discriminant analysis effect size) was performed to detect differentially abundant taxa across groups using the default parameters. Spearman rank correlation tests were performed to evaluate associations between two variables, and *P*-values were adjusted for false discovery rate according to Bonferroni and Hochberg procedure (Benjamini and Hochberg, 1995).

## Results

### Characteristics of the Samples and 16S rRNA Gene Sequence Data

After quality trimming and chimera detection followed sample rarefaction, an average of 45,473 high-quality sequences (range from 47,448 to 54,488) remained. In total, sequences from the cecal microflora could be classified into 43 genera in control group of 6 days (C-6d, *n* = 8), 47 genera in D-GPV infection group of 6 days (I-6d, *n* = 9), 60 genera in control group of 15 days (C-15d, *n* = 10) and 55 genera in D-GPV infection group of 15 days (I-15d, *n* = 10), respectively. Basic information for samples, including accession numbers, sequencing reads, length distribution statistics of sequence, species accumulation curves, the number of taxa observed, and rank abundance curve are reported in Supplementary Table S2 and Supplementary Figure S1.

### D-GPV Infection Reduced the Microbial Diversity and Altered the Structure of Community in the Ceca

**FIGURE 1:**
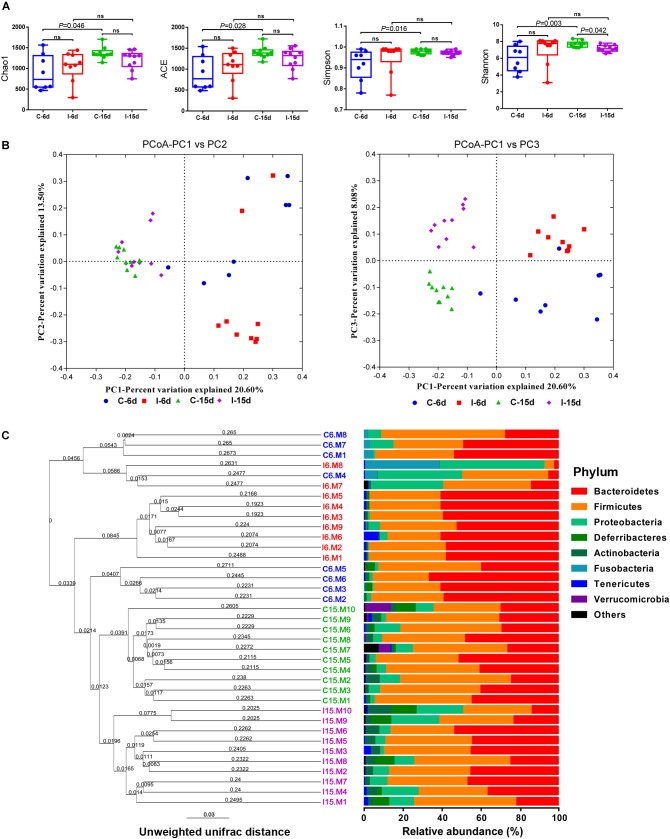
D-GPV infection reduced the microbial diversity and altered the structure of community in the ceca. **(A)** Richness and diversity within caecal microbial from uninfected and D-GPV infected ducks. Values are shown as min to max with the mean value calculated for each groups. Statistical tests were performed using *post hoc* ANOVA and ns indicated no significant difference. **(B)** Principal coordinate analysis of unweighted UniFrac distances. Samples are colored by different group— Blue, Control-6 days (C-6d, *n* = 8); Red, D-GPV infection-6 days (I-6d, *n* = 9); Green, Control-15 days (C-15d, *n* = 10); Purple, D-GPV infection-15 days (I-15d, *n* = 10). Axes are scaled by the percent of variation explained by each principal coordinate. **(C)** Cluster analysis based on unweighted unifrac distance UPGMA. In order to examine the global differences in bacterial composition between the D-GPV infected ducks and the controls over time, we calculated distances between each sample using the unweighted Unifrac. Samples are colored by different group same as **(B)**.

Multiple alpha diversity metrics of richness and diversity revealed obvious distinctions among microbial populations at different post-infection time. At 6 dpi, the sample richness and diversity showed no significant difference (*P* > 0.05) between control group and D-GPV infection group reflected by the abundance index Ace, Chao, and Simpson. However, a significant decrease in diversity reflected by the Shannon analyses from D-GPV infected ducks at 15 dpi (*P* = 0.042) (Figure 1A). Generally, the alpha diversity metrics of richness (Chao1 and ACE) and diversity (Simpson and shannon) in the microbial populations significantly increased (*P* < 0.05) with ducks maturing in the case of uninfected. In contrast, there is no significant difference between the two infection groups with ducks maturing. Collectively, these data suggested that the increase of diversity of the microbial populations was blocked with the extension of D-GPV infection time.

To uncover relationships based on presence or absence of bacterial groups as well as their phylogenetic relatedness, we performed a Principal coordinates (PCoA) analysis of the samples using the phylogenetic-tree-based Unifrac metric. In unweighted UniFrac PCoA, each sample represents a point in multidimensional space based on the composition of the bacterial population in each sample using PC1, PC2, and PC3 (20.6%, 13.50%, and 8.08%, respectively, of the explained variance), and closeness of two points in the PCoA denotes similar bacterial population composition between the samples (Figure 1B). The samples from 6 dpi were separated from 15 dpi on a distinct PC1 axis of variation (left to right), and the separation effect of D-GPV infection groups from control groups on PC3 axis (bottom to top) was better than that of PC2 axis. The analysis showed distinct clustering of microbial communities associated with D-GPV infection and age (or post-infection times), and suggested the distinction between D-GPV infected and control ducks of major shift in the composition of cecal microbiota.

Analysis of similarities (ANOSIM) based on unweighted UniFrac distances for measuring beta-diversity showed that differences among all pairwise comparisons between different groups was statistically significant (*P* < 0.01, AMOVA) (Table 2). The result of samples from D-GPV infection groups clustered differently with those from controls was additionally confirmed by Hierarchical clustering of the samples based on the unweighted pair group method with arithmetic mean (UPGMA) using the unweighted UniFrac (Figure 1C).

**Table 2:** AMOVA *p*-values of treatment groups (permutations = 999).

	C-6d	I-6d	C-15d
I-6d	0.004		
C-15d	0.001	0.001	
I-15d	0.001	0.001	0.001

### D-GPV-Associated Alterations in Cecal Microbiota

**FIGURE 2:**
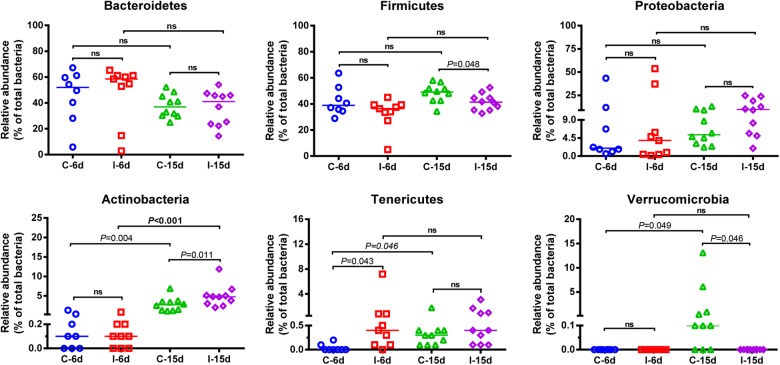
Comparison of the relative abundance of the Bacteroidetes, Firmicutes, Proteobacteria, Actinobactria, Tenericutes, and Verrucomicrobia phyla within groups. Values are shown as a fraction of the total bacteria detected within each samples. Lines represent the median value. Statistical tests were performed using *post hoc* ANOVA and ns indicated no significant difference.

Interestingly, the effects of D-GPV infection on gut microbes varied with growth and development of ducklings. The Bacteroidetes, Firmicutes, and Proteobacteria were the three most dominant phyla detected within caecum of both controls and D-GPV-infection groups (Figure 1C). As noted before, the diversity and spatial structure of gut microbes changed with D-GPV infected period and ages, which were confirmed in microbial composition of phylum level (Figure 2). In the control groups (C-6d and C-15d), there were no significant differences in the major phyla of Bacteroidetes, Firmicutes, and Proteobacteria between 6 dpi and 15 dpi, while the Actinobacteria (*P* = 0.004), Tenericutes (*P* = 0.046) and Verrucomicrobia (*P* = 0.049) were enriched from 6 dpi to 15 dpi, which was consistent with the increase of alpha diversity among control groups. Although there was no difference in alpha diversity between the two infection groups (I-6d and I-15d), the increase relative abundance of Actinobacteria (*P* < 0.001) existed in the course of infection (from 6 dpi to 15 dpi). When compared with control group, D-GPV infection significantly increased the relative abundance of Tenericutes (*P* = 0.043) at 6 dpi and Actinobacteria (*P* = 0.011) at 15 dpi, but decreased the relative abundance of Firmicutes (*P* = 0.048) at 15 dpi. Furthermore, the colonization of Verrucomicrobia was observed in control group at 15 dpi, while there was no Verrucomicrobia in D-GPV infection group. Therefore, the decreased Shannon index was related to the decrease of Firmicutes and Verrucomicrobia during D-GPV infection.

**FIGURE 3:**
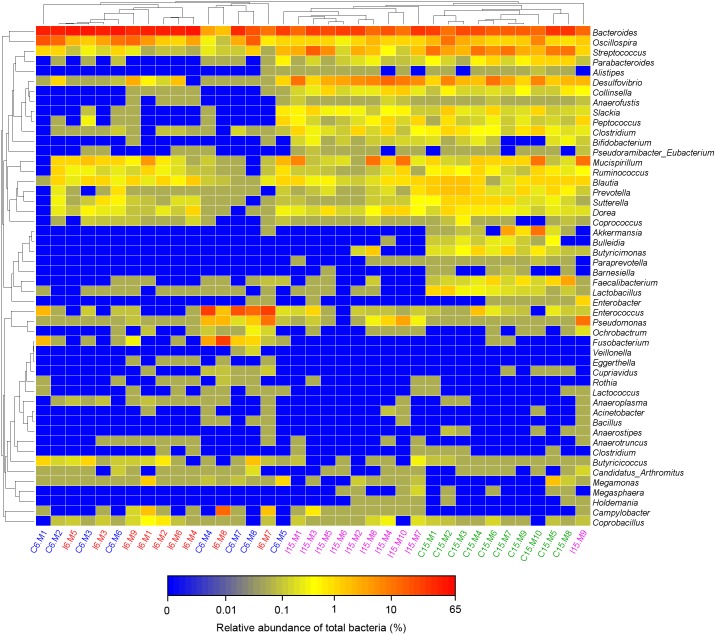
Bacterial distribution of the top 50 abundant genus among 37 samples. Double hierarchical dendrogram shows the bacterial distribution. The heatmap plot depicts the relative percentage of each bacterial genus within each sample. The relative values for bacterial family are indicated by color intensity with the legend indicated under the heatmap.

**FIGURE 4:**
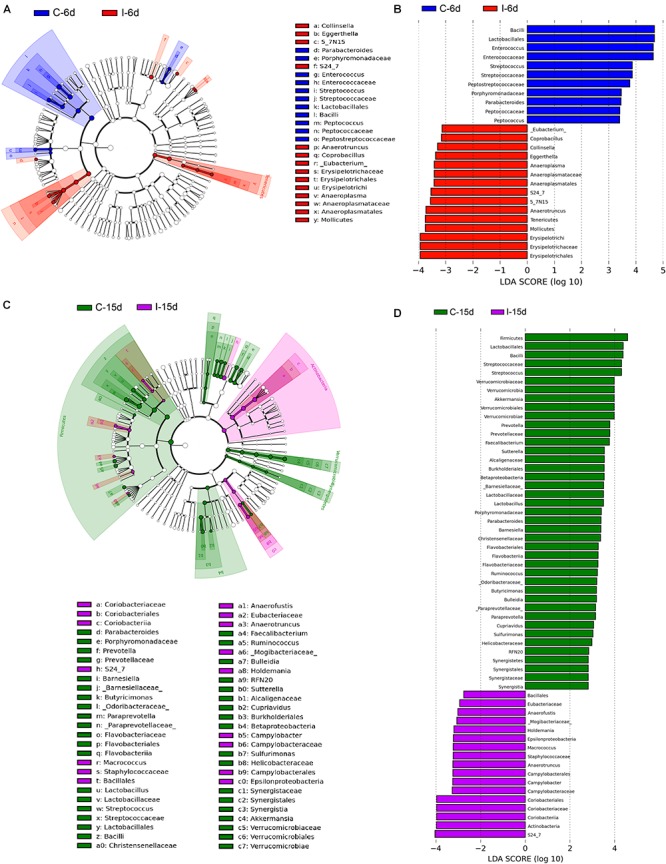
The difference of abundances in taxa between the D-GPV-infected groups and the control groups. **(A)** Taxonomic cladogram obtained from LEfSe sequence analysis at 6 dpi. Biomarker taxa are highlighted by colored circles and shaded areas. Each circle’s diameter reflects the abundance of that taxa in the community. **(B)** The taxa whose abundance differed between the D-GPV-infected samples (I-6d) and the healthy control samples (C-6d) at 6 dpi. The cutoff value of ≥2.0 used for the linear discriminant analysis (LDA) is shown. **(C)** Taxonomic cladogram obtained from LEfSe sequence analysis at 15 dpi. **(D)** The taxa whose abundance differed between the D-GPV-infected samples (I-15d) and the healthy control samples (C-15d) at 15 dpi.

**FIGURE 5:**
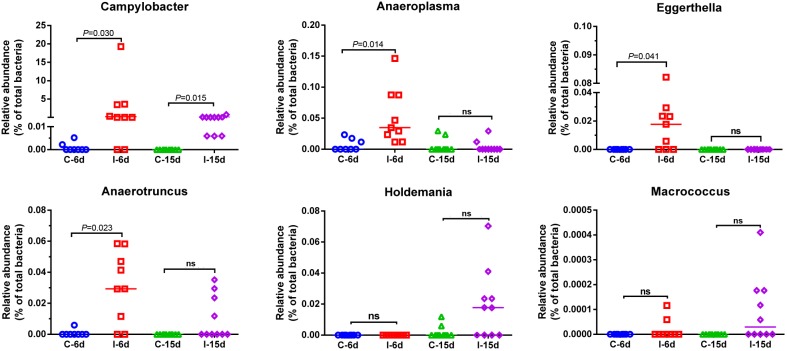
Relative abundance of *Campylobacter*, *Anaeroplasma*, *Eggerthella*, *Anaerotruncus*, *Holdemania*, and *Macrococcus* in the gut microbiota at different post infection time. Values are shown as a fraction of the total bacteria detected within each samples. Lines represent the median value. Statistical tests were performed using *post hoc* ANOVA and ns indicated no significant difference.

Moreover, consistent with beta diversity, clustering analysis of the top 68 genera highlighted differences in their distributions due to D-GPV infection (Figure 3). To identify the specific bacterial taxa associated with D-GPV infection, the linear discriminant analysis (LDA) effect size (LEfSe) method was used to compare the cecal microbiota of healthy controls and D-GPV-infected ducks. The greatest differences in taxa at 6 dpi and 15 dpi between the two communities were, respectively, displayed by the cladogram representative of the structure of cecal microbiota and the predominant bacteria (Figure 4). Some bacteria in the cecal samples sharply varied from the genus classification level. D-GPV infection significantly reduced the relative abundance of genera *Streptococcus*, *Parabacteroides*, *Enterococcus*, and *Pretococcus* (LDA >2) and increased the relative abundance of genera *Anaeroplasma*, *Eggerthella*, *Anaerotruncus*, *Collinsella*, *Coprobacillus*, and *Eubacterium* (LDA >2) compared with control group at 6 dpi (Figure 4A,B). Additionally, the significant decrease relative abundance of genera *Streptococcus*, *Parabacteroides*, *Prevotella*, *Paraprevotella*, *Lactobacillus*, *Barnesiella*, *Butyricimonas*, *Faecalibacterium*, *Ruminococcus*, *Bulleidia*, and *Akkermansia* (LDA >2) and the significantly increased relative abundance of genera *Campylobacter*, *Anaerofustis*, *Anaerotruncus*, *Holdemania*, and *Marcrococcus* (LDA >2) were observed in D-GPV infection group at 15 dpi (Figure 4C,D). These genera contain opportunistic pathogens exhibited enrichment in abundance when infected with D-GPV especially during the early stages of 6 dpi, and there were never detected at high abundances in control samples (Figure 5). Clearly, the D-GPV infection was associated with the aberrant composition of the cecal microbiota, and the effect of D-GPV on the colonization of gut microorganism was more obvious along with the extension of infection time.

### D-GPV Infection Caused Growth Retardation and Decreased the Concentrations of SCFAs

**FIGURE 6:**
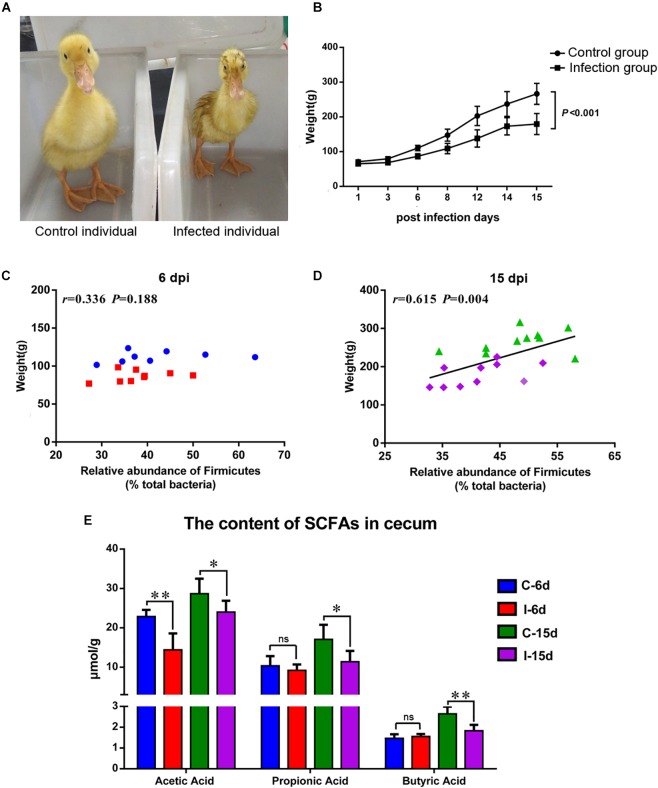
**(A)** Individual comparison between control duck and infected duck. **(B)** Body weight of control group and D-GVP infection group (*P* < 0.01 by two-tailed Student’s *t*-test). **(C,D)** Association between the relative abundance of Firmicutes and body weight of control samples and D-GPV infected samples at 6 dpi and 15 dpi, respectively. Statistical analysis was performed using the Spearman *t*-test. **(E)** Effect of D-GPV infection on SCFAs content in caecum. ^∗^*P* < 0.05, ^∗∗^*P* < 0.01 by *post hoc* ANOVA, and ns meant no significant differences.

The D-GPV-infected ducklings were obvious stunted growth (Figure 6A) and severely underweight compared with the control group (*P* < 0.01) (Figure 6B). Interestingly, no significant association between body weight and the relative abundance of phylum Firmicutes was noted within control and D-GPV infected samples at 6 dpi (*P* = 0.188) (Figure 6C), whereas a trend toward a positive association between body weight and the relative abundance of phylum Firmicutes was observed at 15 dpi (*P* = 0.004) (Figure 6D).

Additionally, lower concentration of SCFAs was detected in the cecal samples collected from D-GPV-infected ducks by HPLC method. The acetic acids were significantly lower in D-GPV-infection groups at both 6 dpi and 15 dpi, furthermore, propionic acid and butyric acids from infection group were reduced at 15 dpi compared to control groups (Figure 6E).

### D-GPV Infection Destroyed the Mucosal Epithelium of the Intestine and Promoted Local Intestinal Inflammation

**FIGURE 7:**
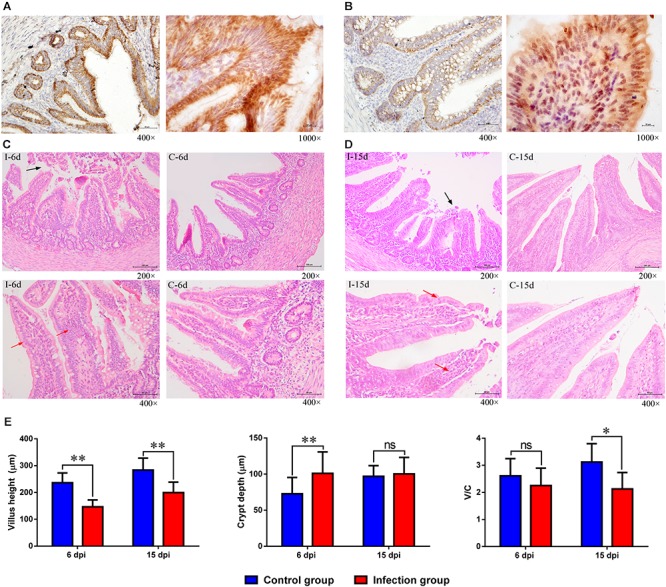
The location of D-GPV antigen and histopathological changes in caecum at 6 dpi and 15 dpi. **(A)** Positive virus signals were detected on the surface of epithelium cells and glandular epithelial cells at 6 dpi. **(B)** Positive virus signals were mainly detected in basal base and basement membrane of cells at 15 dpi. **(C,D)** Histological features in the D-GPV infection group (i) and control group (c) are shown with hematoxylin and eosin staining at 6 dpi and 15 dpi. Necrosis and abscission of mucous epithelial cells are indicated with the black arrow. Proliferation of lymphocytes in laminae propria is indicated with the red arrow. **(E)** Villus height, crypt depth and the ration of villus height to crypt depth (V/C) in caeca of proximal ileum. Values were means ± standard deviation of three independent experiments (^∗^*P* < 0.05, ^∗∗^*P* < 0.01 by *post hoc* ANOVA), and ns meant no significant differences.

The distribution of the immunoreactivity for the D-GPV antigen was confirmed, as determined in paraformaldehyde-fixed, paraffin-embedded tissue sections by IHC. The D-GPV antigen was mostly strongly distributed in mucosal epithelium and glandular epithelial cells. The D-GPV antigen was randomly and widely distributed on the surface of epithelia at 6 dpi (Figure 7A). Nevertheless, the antigen was mainly distributed in basal region and basement membrane of epithelial cells and cells of laminae propria at 15 dpi (Figure 7B). Positive virus signals were not detected in control samples (Supplementary Figure S2).

Cecal histopathology analysis showed that necrosis and abscission of mucosal epithelial cells were observed in the 9/10 of D-GPV infected samples at 6 dpi and 6/10 of D-GPV infected samples at 15 dpi, as well as 10/10 of infected samples at 6 dpi and 9/10 of infected samples at 15 dpi showed local lymphocytic infiltration in chorioepithelium and laminae propria (Figure 7C,D). Importantly, the villus height of D-GPV infected samples was significantly decreased (*P* < 0.01) at 6 dpi and 15 dpi, and the crypt depth of D-GPV infected samples was significantly increased (*P* < 0.01) at 6 dpi (Figure 7E). The ratio of villus height/crypt depth was significantly reduced at 15 dpi (*P* < 0.05) in the ceca of proximal ileum (Figure 7E). The decreased ratio of height of villus and villus height/crypt depth reduced the absorptive area of the intestine and the mature cell manifold, which suggested that D-GPV weaken the absorbing ability of ceca.

**FIGURE 8:**
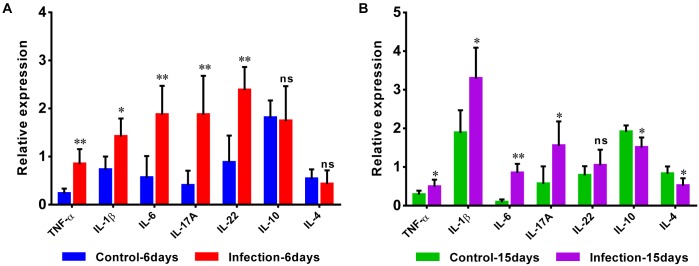
Quantitative RT-PCR analysis of the expression of pro-inflammatory TNF-α, IL-1β, IL-6, IL-17A, and IL-22, as well as anti-inflammatory IL-10 and IL-4 in the caecum from control and D-GPV infection groups at 6 dpi **(A)** and 15 dpi **(B)**.

Additionally, the expansion of pro-inflammatory cytokines was observed in D-GPV infection group. The mRNA expressions of pro-inflammatory TNF-α, IL-1β, IL-6, IL-17A, and IL-22 were significantly up-regulated after D-GPV infection, although the expression of IL-22 had no significant difference at 15 dpi. While the mRNA expression of anti-inflammatory IL-10 and IL-4 were down-regulated at 15 dpi (Figure 8A,B).

### Relationships Between Cecal Microbiota and the Relative Expression of Cytokines

**FIGURE 9:**
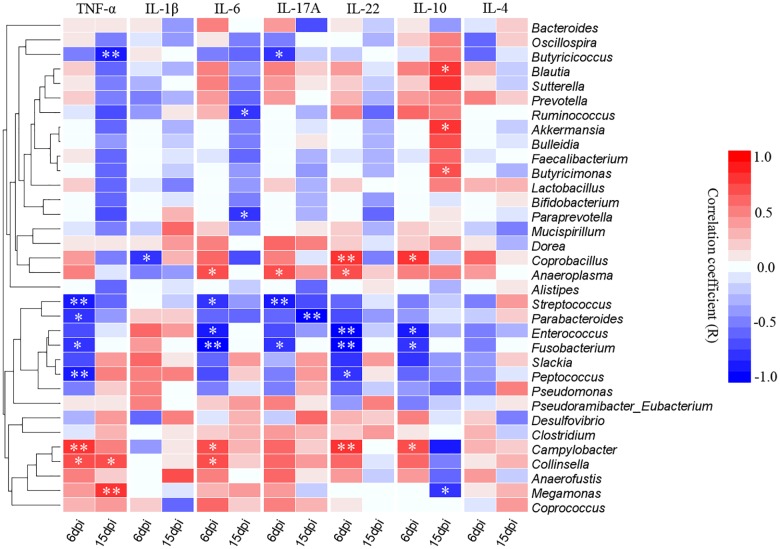
Correlogram showing Spearman’s correlations between bacterial genera and cytokine responses in the caecum. Heatmap representing positive (blue shading) and negative (red shading) associations between pro-inflammatory TNF-α, IL-1β, IL-6, IL-17A, and IL-22 and anti-inflammatory IL-10 and IL-4. Only the predominant bacterial genera (relative abundance ≥0.01% in every sample at least one group) for which abundance was significantly associated with inflammatory cytokines expression are presented; Clustering was performed based on genera associations with the inflammatory cytokines. *P*-values were adjusted for false discovery rate according to Bonferroni and Hochberg procedure. An asterisk indicates a significance correlation between the bacterial taxa and the cytokine in a tissue (^∗∗^*P* < 0.01, ^∗^*P* < 0.05).

The correlative relationships among pro-inflammatory and anti-inflammatory cytokine expression and cecal predominant bacterial populations were evaluated in this study (Figure 9). The result showed that the pro-inflammatory TNF-α was significantly and negatively correlated with Streptococcus, Parabacteroides, Fusobacterium and Peptococcus at 6 dpi and also negatively correlated with Butyricicoccus at 15 dpi, while it was significantly and positively correlated with Campylobacter and Collinsella at 6 dpi as well as Collinsella and Megamonas at 15 dpi. Expression of pro-inflammatory IL-1β was significantly and negatively correlated with Collinsella at 6 dpi. Expression of IL-6 was significantly and negatively correlated Streptococcus, Enterococcus and Fusobacterium at 6 dpi as well as Ruminococcus and Prevotella at 15 dpi, however, it was positively associated with Anaeroplasma, Campylobacter and Collinsella at 6dpi. The pro-inflammatory IL-17A was negatively associated with Butyricicoccus, Streptococcus and Fusobacterium while correlated positively with Anaeroplasma at 6 dpi, as well as the Parabacteroides was significantly and negatively correlated with the expression of IL-17A at 15 dpi. The expression of IL-22 correlated negatively with Enterococcus, Fusobacterium and Peptococcus while positively correlated with *Coprobacillus*, Anaeroplasma, and Campylobacter at 6 dpi. The expression of anti-inflammatory IL-10 correlated positively with *Blautia*, *Akkermansia*, and *Butyricimonas* while it was significantly and negatively correlated with Megamonas at 15 dpi.

## Discussion

The gut microbiota has profound influences on the normal structural and functional development of the mucosal immune system (Hooper et al., 2012). The poultry gut is important to health, however, little is known about how the complex gut microbiota are affected during viral infection. This study first analyzes the effect of D-GPV infection on gut microbiota of Cherry Valley duck, and provides a new perspective for the study on the waterfowl infectious diseases.

In this study, the caecum was utilized to analyze the gut microbiota, for the role in digestion and the overall health of birds. Based on a preliminary analysis of alpha diversity and beta diversity, all the diversity metrics of microbial populations increased significantly as healthy ducks matured. However, the alpha diversity of the gut microbiota in D-GPV infected ducks was decreased, and the structure and composition of cecal microbiota were different from control ducks. The Bacteroidetes, the Firmicutes, and the Proteobacteria were major bacterial phyla in cecal microbiota of Cherry Valley ducks, as previously described in Pekin ducks (Best et al., 2016). The two most dominant phyla of Firmicutes and Bacteroidetes in different organisms such as birds or mammals play an important role in intestinal microbiota metabolism in most studies (Ley et al., 2008; Kohl, 2012). The Firmicutes, a major phylum in the intestines or feces of many animals, is related to the digestibility of crude forage (Mohd Shaufi et al., 2015). In our study, the D-GPV-infected ducklings showed slight weakness, a loss of appetite, and loose stool excretion. We found that the D-GPV was mainly targeted in intestinal epithelial cells with necrosis and abscission of intestinal epithelial cells and lymphocytic infiltration. Importantly, the shorter intestinal villus and the increased crypt depth in D-GPV infected samples suggested intestinal growth retardation. In addition, a significant positive correlation between body weight and the relative abundance of Firmicutes was observed at 15 dpi. Therefore, we speculated that the intestinal injury and the decreased relative abundance of Firmicutes would inevitably decrease the digestion and absorption of nutrients, which was an important causation of growth retardation and diarrhea in Cherry Valley ducks after D-GPV infection.

Interestingly, we demonstrated a significant perturbation in colonization of SCFAs-producing bacteria during D-GPV infected Cherry Valley ducks. This perturbation was characterized by the decreased relative abundance of the dominance, such as the genus *Streptococcus*, *Faecalibacterium*, *Lactobacillus*, *Enterococcus*, *Bulleidia*, and *Ruminococcus* in Firmicutes phylum and the genus *Parabacteroides*, *Prevotella*, and *Paraprevotella* in Bacteroidetes phylum, accompanying with a decrease in microbial diversity. Meanwhile, D-GPV infection obstructed the colonization of some new bacteria at 15 dpi, for instance, the genus *Paraprevotella*, *Bulleidia*, *Akkermansia*, *Butyricimonas*, and *Barnesiella* (in Supplementary Figure S3). Thereinto, the genus *Streptococcus* was a primary acetic acid producer, and the *Lactobacillus*, *Enterococcus*, and *Parabacteroide* were one of the lactic acid and propionic acid producing bacteria, while the genera *Butyricimonas*, *Prevotella*, *Ruminococcus*, and *Faecalibacterium* were the major butyric acids producing bacteria. The decreased content of SCFAs was also found in D-GPV infected samples. Accordingly, we speculated that the reduction of SCFAs might be linked to the alterations of SCFAs-producing bacteria in cecal microbiota, which were important members of the endogenous bacteria playing an anti-inflammatory role in intestinal tract (Ménard et al., 2004; Fedorova and Danilenko, 2014; Nicholas and Rudensky, 2014). The interaction of SCFAs and G-protein-coupled receptors (GPR) is one of the important signal between gut microbiota and immune system for regulating the homeostasis and maintaining the balance between immune tolerance to commensals bacteria and immunity to pathogens (Kim et al., 2016). There is now sufficient evidence to indicate that SCFAs play an important role in the maintenance of health and the development of disease. It has been reported that SCFAs can enhance the host defenses against pathogens by activating the G-protein-coupled receptors (GPR) to mediate Regulatory T cell (Tregs) development and induce T helper differentiation (Arpaia et al., 2013; Smith et al., 2013). In addition, SCFAs such as butyrate and propionate suppress the activation of nuclear factor-kappaB (NF-κB) via GPR109A or GPR43 receptors to modulate the gene expression of inflammatory cytokines and suppress gut inflammation (Inan et al., 2000; Singh et al., 2014). Therefore, the decreases of SCFAs and SCFAs-producing bacteria could promote intestinal inflammatory injury in the process of D-GPV infection.

In addition, some opportunistic pathogens increased in abundance as the disease progressed, including the genera *Campylobacter*, *Anaerofustis*, *Anaeroplasma*, *Eggerthella*, *Anaerotruncus*, *Holdemania*, *Macrococcus*, *Coprobacillus*, *Collinsella*, and *Eubacterium* present within D-GPV infected ducks especially during the early stages of 6 dpi. Thereinto, the enrichment of *Coriobacteriaceae*, *Coprobacillus*, and *Mogibacteriaceae* have been reported in colorectal cancer of human (Chen et al., 2012; Gao et al., 2014). Furthermore, the genus *Campylobacter* and *Eggerthella* were reported to be associated with intestinal inflammation (Spiller et al., 2000; Lau et al., 2004). It was indicative to determine whether the increased abundance of D-GPV infection-related genera could serve as a predisposing cause of gastrointestinal abnormalities symptoms and threat to the health of host. Infiltration of inflammatory cells in multiple organs and decrease of lymphocytes in immune organs have been both reported in D-GPV-infected Cherry valley ducks and mule ducks (Chen H. et al., 2016; Liu et al., 2018). The expansion of the pro-inflammatory cytokines TNF-α, IL-1β, IL-6, IL-17A, and IL-22 is important for the immune responses of infectious individuals (Neurath, 2015; Mcdermott et al., 2016). Furthermore, IL-17A participates in the primary responses to fungi and also bacterial infections and induction of the pro-inflammatory based diseases (Douzandeh-Mobarrez and Kariminik, 2017). TNF-α, IL-1β, IL-6, IL-17A, and IL-22, as the most important pro-inflammatory cytokines, were largely up-regulated in our experimental process of D-GPV infection which played an important role to induce inflammation and the recruitment of leukocytes.

In the correlation analyses, the abundance of SCFAs-producing bacteria such as *Streptococcus*, *Parabacteroides*, *Enterococcus*, *Fusobacterium* and *Peptococcus* were negatively associated with the pro-inflammatory TNF-α, IL-6, IL-17A, and IL-22, in particularly, the anti-inflammatory IL-10 was positively related with some butyric acid producing bacteria such as *Blautia* and *Butyricimonas* at 15 dpi. Nevertheless, the opportunistic pathogens such as *Anaeroplasma*, *Campylobacter* and *Collinsella* were positively associated with TNF-α, IL-6, IL-17A, and IL-22. Interestingly, it appears that altered population of gut microbiota can be associated with pathologic expression of inflammatory factor. For example, Kamiya et al. revealed that oral administration of SCFAs-producing bacteria (*Lactobacillus bulgaricus* and *Streptococcus thermophiles*) results in up-regulation of IL-17A by the Peyer’s patches resident T lymphocytes (Kamiya et al., 2016). Chen J. et al. (2016) also showed the abundance of *Collinsella* is associated with pathologic expression of IL-17A and deterioration of rheumatoid arthritis. Furthermore, the high levels of *Campylobacter jejuni* leads to alteration in gut microbiota population and expression of IL-17A, IL-22, and TNF-α in pathologic format (Heimesaat et al., 2016; Rodrigues et al., 2018). A feature of IBD-ulcerative colitis and Crohn’s disease was a change in ’healthy’ microbiota such as *Bifidobacterium* and *Bacteriodes*, and a concurrent reduction in SCFAs (Hudafaujan et al., 2010; Chaysavanh et al., 2012). Accordingly, the increase of pro-inflammatory cytokines and decrease of anti-inflammatory cytokines were associated with the decrease of SCFAs-producing bacteria and the enrichment of opportunistic pathogens which could promote the development of intestinal inflammation in D-GPV infection.

The effect on the gut microbiome have been reported in some immunosuppressive virus, such as HIV in human (Lozupone et al., 2013), Marek’s disease virus (MDV) in chicken (Perumbakkam et al., 2014) and canine distemper virus (CDV) in giant panda (Zhao et al., 2017). CD4+ lymphocytes have been reported to help regulate the growth of bacteria within the gut microbiome (Slack et al., 2009). Although the decreased activity of CD4+ lymphocytes is one of the cause of affecting gut microbiota composition and diversity, the function of dendritic cells in using MHC-II to present antigens to activate T cells for sampling gut microbes is mentionable to recognition and regulation of gut microbes (Farache et al., 2013; Bolnick et al., 2015). It is essential in regulating the gut microbiota that adaptive cellular immunity has the ability to modulate flexible responses to host-specific microbial communities by inducing inflammatory immune attacks or tolerance via anti-inflammatory pathways (Sun et al., 2015). It is reported that MHC are associated with GPV infection, and MHC gene is the susceptible gene to GPV (Zhu et al., 2015). To some extent, the alterations of the gut microbiota support the statement that the immune system has a pivotal role in shaping the composition of gut microbiota. Nevertheless, there is no clear evidence that whether D-GPV infection alters the gut microbiota through the suppressing activity of CD4+ lymphocytes or regulating the gene expression of MHC-II. Therefore, the interaction between intestinal mucosal immune response and gut microbiota during D-GPV infection needs further investigation.

## Conclusion

In conclusion, the present study provides a new insight for dysbiosis caused by waterfowl parvovirus on the host gut microbiome. D-GPV targeted intestinal epithelial cells and caused the dysbiosis in gut microbiota accompanying with decrease in the content of short chain fatty acids in ceca. Moreover, the results show a correlation among the deletion of some specific SCFAs-producing bacteria, the enrichment of opportunistic pathogens and intestinal inflammatory injury in D-GPV infection. This research sheds light into the correlative new field in the mechanism of D-GPV and a theoretical basis for dysbiosis of this disease.

## Author Contributions

JX, CH, and ZC designed the experiments. JX, DC, and WL performed the experiments. JX and QL analyzed the data. JX, QL, XL, and DC drafted the manuscript. AC, LT, JF, YO, YG, and WW participated in data interpretation and manuscript editing. All authors reviewed the manuscript and finally approved the publication of the article.

## Conflict of Interest Statement

The authors declare that the research was conducted in the absence of any commercial or financial relationships that could be construed as a potential conflict of interest.
