# Supplementary material for: Impacts of Duck-Origin Parvovirus Infection on Cherry Valley Ducklings From the Perspective of Gut Microbiota
Source: Front Microbiol. 2019 Mar 28;10:624. doi: 10.3389/fmicb.2019.00624 (PMC6450226; doi:10.3389/fmicb.2019.00624)
Supplement: Supplementary file 1 [file Data_Sheet_1.docx]

Supplementary Material

Impacts of Duck-origin Parvovirus infection on the Cherry Valley ducklings from the perspective of gut microbiota

**Qihui Luo^1,2#^, Jing Xu^1,2#^, Chao Huang^1,2#^,** **Xinyu Lei^1,2^, Dongjing Cheng^1,2^, Wentao** **Liu^1,2^,** **Anchun Cheng^1^, Li Tang^1,2^, Jing Fang^1^, Yangping Ou^1^, Yi Geng^1^, Wenxiu Wang^3^, Zhengli Chen^1,2*^**

*** Correspondence:** Zhengli Chen: chzhli75@163.com

# Supplementary Data

Materials and Methods

**Bacterial DNA Isolation.** Total bacterial genomic DNA samples were extracted using the Fast DNA SPIN extraction kits (MP Biomedicals, Santa Ana, CA, USA), following the manufacturer’s instructions, and stored at −20°C prior to further analysis. The quantity and quality of extracted DNAs were measured using a NanoDrop ND-1000 spectrophotometer (Thermo Fisher Scientific, Waltham, MA,USA) and agarose gel electrophoresis, respectively.

**16S rDNA Amplicon Sequencing.** PCR amplification of the bacterial 16S rRNA genes V3–V4 region was performed using the forward primer 338F (5’-ACTCCTACGGGAGGCAGCA-3’) and the reverse primer 806R (5’-GGACTACHVGGGTWTCTAAT-3’). Sample-specific 7-bp barcodes were incorporated into the primers for multiplex sequencing. The PCR components contained 5 μl of Q5 reaction buffer (5×), 5 μl of Q5 High-Fidelity GC buffer (5×), 0.25 μl of Q5 High-Fidelity DNA Polymerase(New England Biolabs, Inc., USA) (5U/μl), 2 μl (2.5 mM) of dNTPs, 1 μl (10 uM) of each Forward and Reverse primer, 2 μl of DNA Template, and 8.75 μl of ddH_2_O. Thermal cycling consisted of initial denaturation at 98 °C for 2 min, followed by 25 cycles consisting of denaturation at 98 °C for 15 s, annealing at 55 °C for 30 s, and extension at 72 °C for 30 s, with a final extension of 5 min at 72 °C. PCR amplicons were purified with Agencourt AMPure Beads (Beckman Coulter, Indianapolis, IN) and quantified using the PicoGreen dsDNA Assay Kit (Invitrogen, Carlsbad, CA, USA). After the individual quantification step, amplicons were pooled in equal amounts, and pair-end 2×300 bp sequencing was performed using the Illlumina MiSeq platform with MiSeq Reagent Kit v3 at Shanghai Personal Biotechnology Co., Ltd (Shanghai, China).

**Sequence Analysis.** The Quantitative Insights Into Microbial Ecology (QIIME, v1.8.0) pipeline was employed to process the sequencing data, as previously described(Caporaso et al., 2010). Briefly, raw sequencing reads with exact matches to the barcodes were assigned to respective samples and identified as valid sequences. The low-quality sequences were filtered through following criteria(Gill and Nelson, 2006; Chen and Jiang, 2014): sequences that had a length of < 150 bp, sequences that had average Phred scores of < 20, sequences that contained ambiguous bases, and sequences that contained mononucleotide repeats of > 8 bp. Paired-end reads were assembled using FLASH(Magoč and Salzberg, 2011). Chimera sequences were deleted with QIME Software (Quantitative Insights Into Microbial Ecology, v1.8.0, <http://qiime.org/>). After chimera detection, the remaining high-quality sequences were clustered into operational taxonomic units (OTUs) at 97 % sequence identity by UCLUST(Edgar, 2010). A representative sequence was selected from each OTU using default parameters. OTU taxonomic classification was conducted by BLAST searching the representative sequences set against the Greengenes Database(Desantis et al., 2006) using the best hit(Altschul et al.). An OTU table was further generated to record the abundance of each OTU in each sample and the taxonomy of these OTUs. OTUs containing less than 0.001 % of total sequences across all samples were discarded. To minimize the difference of sequencing depth across samples, an averaged, rounded rarefied OTU table was generated by averaging 100 evenly resampled OTU subsets under the 90 % of the minimum sequencing depth for further analysis.

# Supplementary Tables and Figures

# Supplementary Table 1. Detailed composition of commercial forage.

| **Ingredient** | **content** |
| --- | --- |
| corn | 40% |
| canola meal | 20% |
| stalkforage | 10% |
| broken rice | 10% |
| bran | 10% |
| fish protein | 7.5% |
| digested tankage | 7.5% |
| shell powder | 1% |
| salt | 0.5% |

**Supplementary Table 2.** **Accession numbers and effective sequence reads through quality screening.**

| **Sample name** | **Accession number** | **Sequencing reads** |
| --- | --- | --- |
| C6.M1 | SRR8491580 | 41261 |
| C6.M2 | SRR8491579 | 43176 |
| C6.M3 | SRR8491582 | 48916 |
| C6.M4 | SRR8491581 | 36039 |
| C6.M5 | SRR8491584 | 44366 |
| C6.M6 | SRR8491583 | 42858 |
| C6.M7 | SRR8491586 | 43279 |
| C6.M8 | SRR8491585 | 49758 |
| I6.M1 | SRR8491578 | 49882 |
| I6.M2 | SRR8491577 | 54488 |
| I6.M3 | SRR8491570 | 41330 |
| I6.M4 | SRR8491569 | 48243 |
| I6.M5 | SRR8491568 | 41200 |
| I6.M6 | SRR8491567 | 43535 |
| I6.M7 | SRR8491574 | 39502 |
| I6.M8 | SRR8491573 | 48518 |
| I6.M9 | SRR8491572 | 46328 |
| C15.M1 | SRR8491557 | 53566 |
| C15.M2 | SRR8491550 | 51323 |
| C15.M3 | SRR8491551 | 50324 |
| C15.M4 | SRR8491561 | 45024 |
| C15.M5 | SRR8491560 | 43695 |
| C15.M6 | SRR8491563 | 48989 |
| C15.M7 | SRR8491562 | 44084 |
| C15.M8 | SRR8491565 | 45275 |
| C15.M9 | SRR8491564 | 42646 |
| C15.M10 | SRR8491566 | 48535 |
| I15.M1 | SRR8491571 | 47448 |
| I15.M2 | SRR8491576 | 49371 |
| I15.M3 | SRR8491575 | 47969 |
| I15.M4 | SRR8491554 | 49324 |
| I15.M5 | SRR8491555 | 49177 |
| I15.M6 | SRR8491552 | 45784 |
| I15.M7 | SRR8491553 | 49311 |
| I15.M8 | SRR8491558 | 47826 |
| I15.M9 | SRR8491559 | 44718 |
| I15.M10 | SRR8491556 | 50694 |

**Supplementary** **Figure 1.** (A) Length distribution statistics of sequencing reads. (B) Species accumulation curves of Specaccum. (C) The number of taxa observed in every sample. (D) Rank abundance curve.


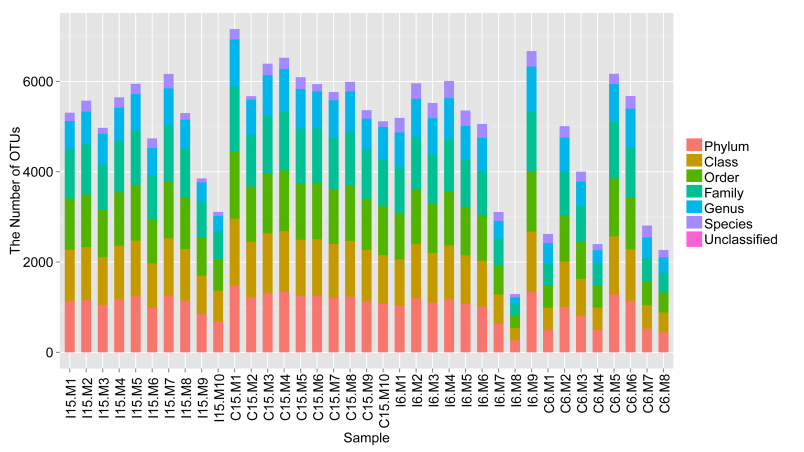

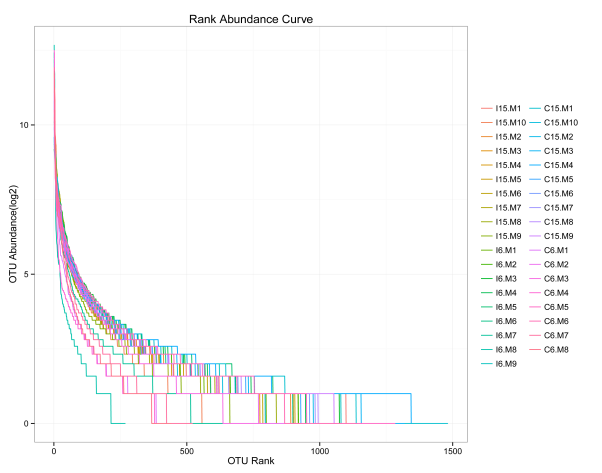

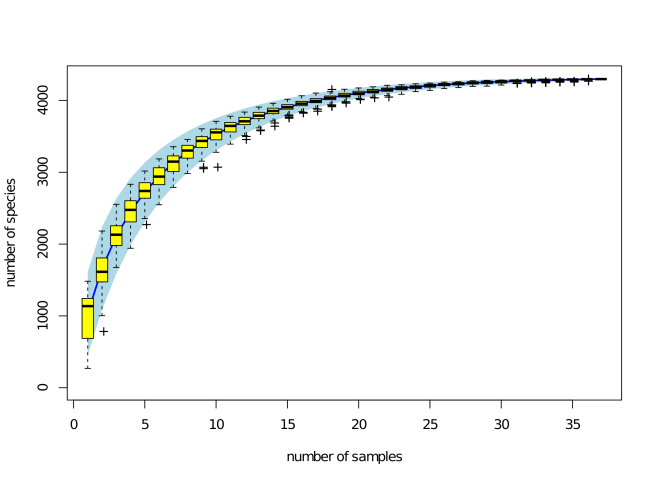

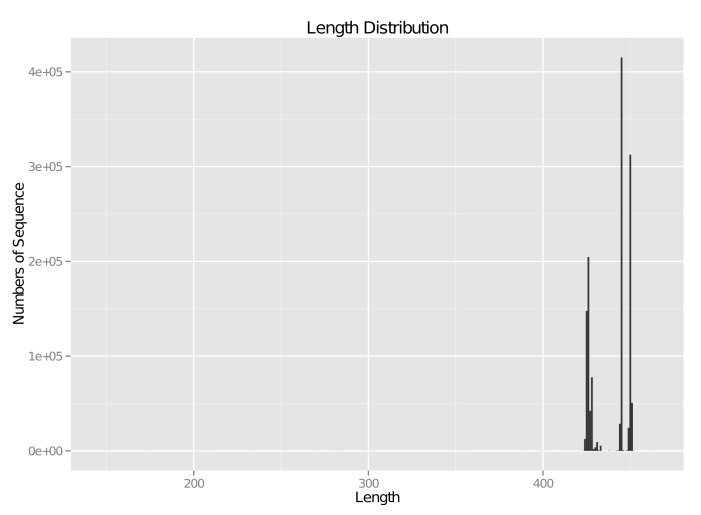


A

B

C

D

**Supplementary Figure 2. Negative controls of** **immunohistochemical and histopathological analyses.** Positive virus signals were not detected in control samples.

**
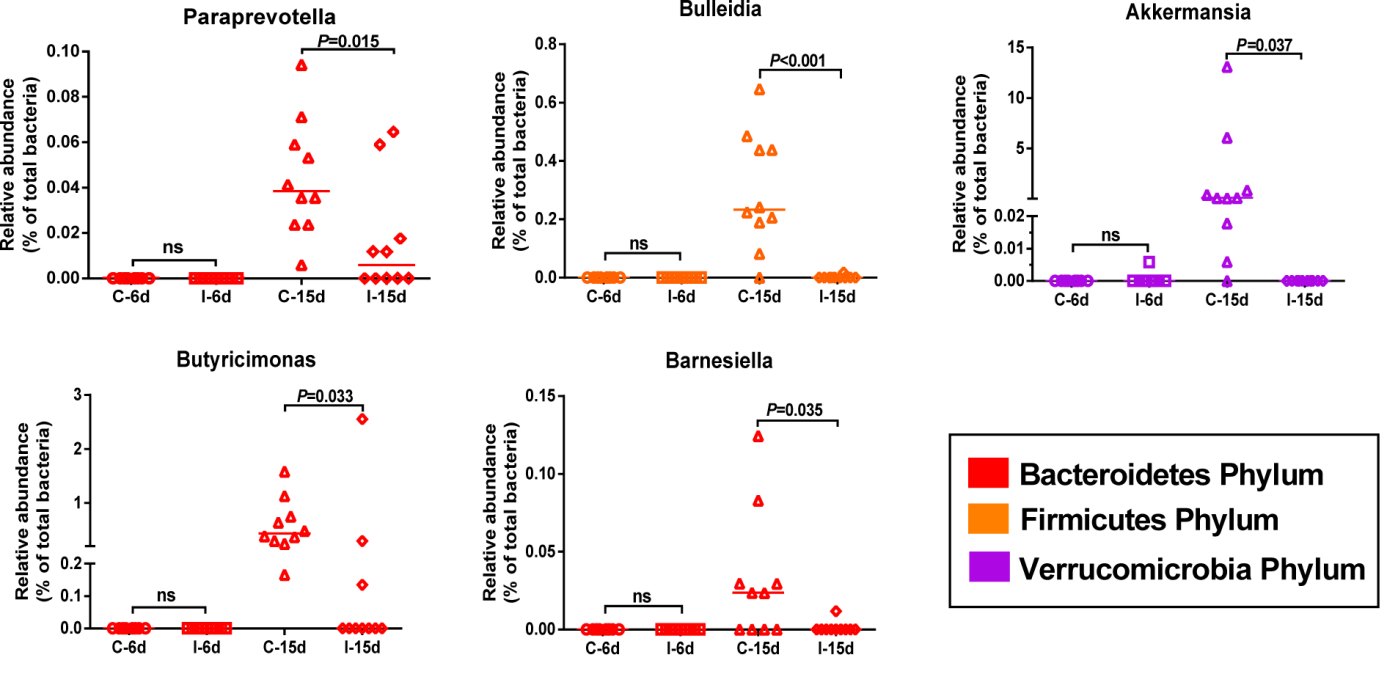
****Supplementary Figure 3.** **Decrease in the abundance of bacteria** **from disease-associated genera** **in the D-GPV-infected ducks.** Plots showing individual relative abundance of various genera that were statistically different between control and D-GPV infected ducks. Values are shown as a fraction of the total bacteria detected within each individual. Lines represent the median value. Statistical tests were performed using post-hoc ANOVA and ns indicated no significant difference.

**Supplementary References**

Altschul, S.F., Madden, T.L., Schäffer, A.A., Zhang, J., Zhang, Z., Miller, W., et al. Gapped BLAST and PSI-BLAST: a new generation of protein database search programs.

Caporaso, J.G., Kuczynski, J., Stombaugh, J., Bittinger, K., Bushman, F.D., Costello, E.K., et al. (2010). QIIME allows analysis of high-throughput community sequencing data.

Chen, H., and Jiang, W. (2014). Application of high-throughput sequencing in understanding human oral microbiome related with health and disease. *Frontiers in Microbiology* 5(508)**,** 508.

Desantis, T.Z., Hugenholtz, P., Larsen, N., Rojas, M., Brodie, E.L., Keller, K., et al. (2006). Greengenes, a chimera-checked 16S rRNA gene database and workbench compatible with ARB. *Applied & Environmental Microbiology* 72(7)**,** 5069-5072.

Edgar, R.C. (2010). Search and clustering orders of magnitude faster than BLAST. *Bioinformatics* 26(19)**,** 2460.

Gill, S.R., and Nelson, K.E. (2006). et al., “Metagenomic analysis of the human distal gut microbiome. *Science* 312(5778)**,** 1355-1359.

Magoč, T., and Salzberg, S.L. (2011). FLASH: fast length adjustment of short reads to improve genome assemblies. *Bioinformatics* 27(21)**,** 2957-2963.
